# A framework for evaluating biosafety and biosecurity in national network of biosafety level-3 laboratories in India: an initiative under national one health mission

**DOI:** 10.3389/fbioe.2025.1611648

**Published:** 2026-02-19

**Authors:** Deepak Y. Patil, Rima R. Sahay, Anita Shete, Anoop Velayudhan, Harmanmeet Kaur, Archana Upadhyay, Pradip Barde, Gururaj R. Deshpande, Uma Prajwal Nalavade, Shailesh D. Pawar, Madhuri Kanitkar, Nivedita Gupta, Pragya Dhruv Yadav, Members of the National network of BSL-3 laboratories

**Affiliations:** 1 National Institute for One Health, Nagpur, Maharashtra, India; 2 Indian Council of Medical Research-National Institute of Virology, Pune, Maharashtra, India; 3 Academy of Scientific and Innovative Research (AcSIR), Ghaziabad, Uttar Pradesh, India; 4 Indian Council of Medical Research, New Delhi, India; 5 Jabalpur Zonal Unit, Indian Council of Medical Research-National Institute of Virology, Jabalpur, Madhya Pradesh, India; 6 Mumbai Unit, Indian Council of Medical Research-National Institute of Virology, Mumbai, Maharashtra, India; 7 Maharashtra University of Health Sciences, Nashik, Maharashtra, India

**Keywords:** biosafety, biosecurity, BSL-3 laboratory network, India, one health

## Abstract

Due to emergence and re-emregence of various infectious diseases across human, animal, wildlife, and environmental sector, there is rapid expansion of high containment Biosafety level 3 (BSL-3) laboratories in India under National One Health Mission. Although, the strong regulatory framework for BSL-3 laboratories exists in India, there is no standard tool to assess the compliance of these laboratories to biosafety and biosecurity parameters, assess staff competencies, sample archival & disposal and reporting processes. In view of this, the critical need was realized to develop a standard assessment tool to periodically assess the performance of BSL-3 laboratories. The tool includes specific sections with reference to a scoring checklist that assesses staff and training, sample handling and transportation, sample processing and testing procedures, data management and reporting, biomedical waste management, emergency preparedness and response, as well as general biosafety. The tool has been developed based on the strategies of the DBT-ICMR guidelines for establishment and certification of BSL-3 laboratories, International Health Regulations and Global Health Security Agenda. It is aimed to evaluate the preparedness of BSL-3 laboratories for safe and prompt testing during outbreaks ensuring the standards of biosafety and biosecurity are followed with the efficient sample processing and reporting. It has a potential to be adopted and used globally by various BSL-3 laboratories and auditors across the world.

## 1 Introduction

With the growing threat of emerging/re-emerging and zoonotic infectious diseases, the critical importance of high containment laboratories has been realized. Biosafety level 3 (BSL-3) laboratories are needed to handle and test samples from any outbreak of unknown or novel origin. Considering the growing demand of BSL-3 laboratories in human, animal, wildlife, and environmental sectors, different government departments in India have embarked on building of BSL-3 laboratories to accommodate their specific specialized needs. This led to rapid expansion of BSL-3 laboratories with varying human resource capabilities, in various parts of the country, thereby increasing the biosafety and biosecurity concerns.

Recently, the Government of India, under the National One Health Mission (NOHM) has developed a plan to repurpose various BSL-3 laboratories in the country for work across different one health sectors. An interdepartmental National BSL-3 laboratory network has been established which includes laboratories from all the above sectors. Twenty two laboratories included in this network have recieved trainings from apex laboratories [Indian Council of Medical Research-National Institute of Virology (ICMR-NIV), Pune, Maharashtra and Indian Council of Agricultural Research-National Institute for High Security Animal Diseases (ICAR-NIHSAD), Bhopal, Madhya Pradesh] for undertaking testing of samples from human, animal, wildlife and environmental origin, and are expected to provide laboratory support to all sectors in times of outbreaks, irrespective of their original domain of work. This initiative is aimed at ensuring optimum use of the existing BSL-3 laboratories across different geographical locations for quick outbreak responses and restrict the irrational establishment of such laboratories across various sectors by creating a laboratory sharing and redeployment model. Considering the growing threat of emerging and reemerging diseases, the laboratories which have mainly worked on research component are now mandated to provide laboratory diagnostic support during public health emergency in future. Hence, it is critical to monitor their core competence and laboratory practices to provide safe and quick sample processing and reporting.

**FIGURE 1:**
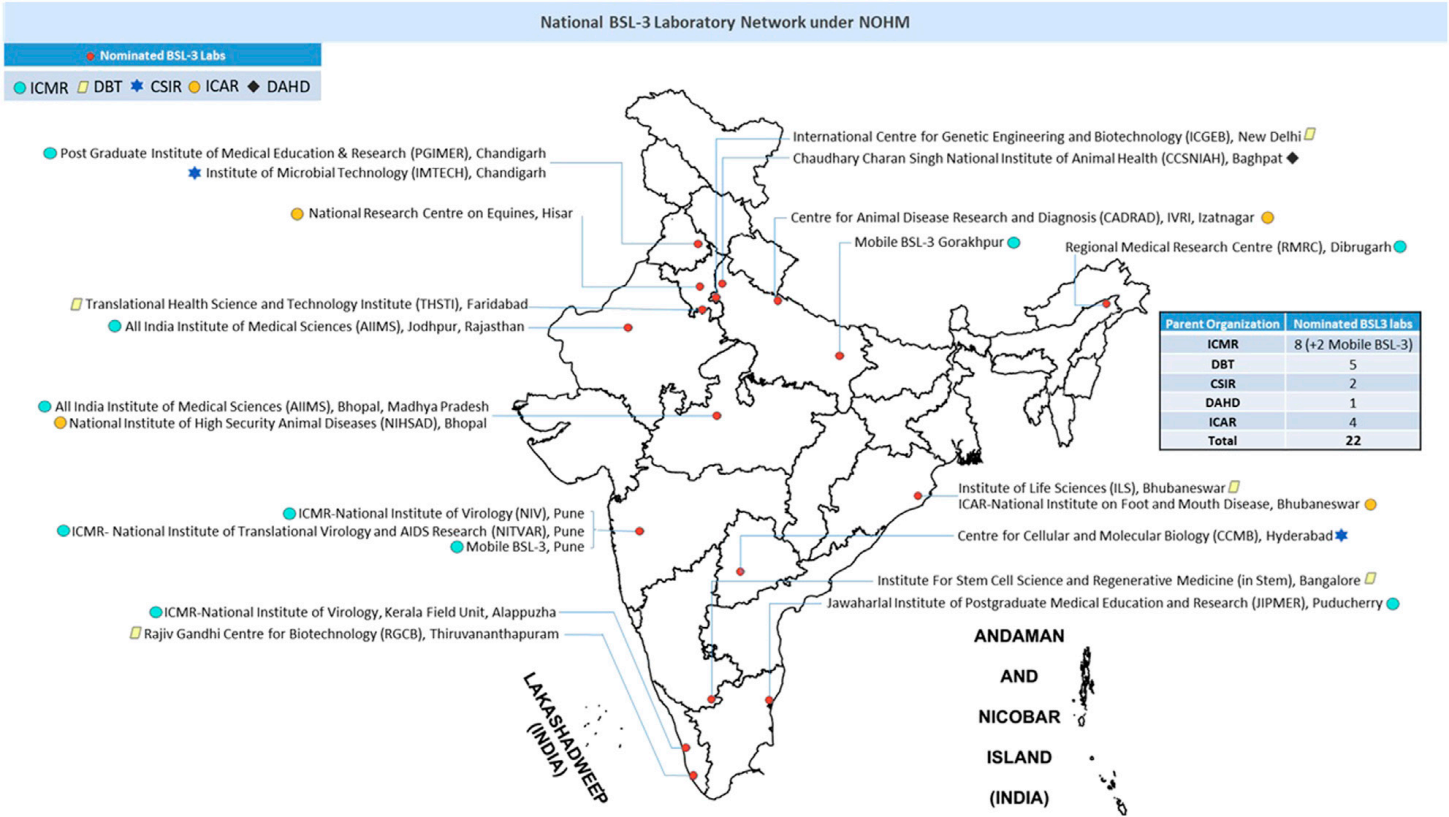
National network of BSL-3 laboratories in India. ICMR, indian council of medical research; DBT, department of biotechnology; CSIR, council of scientific and industrial research; ICAR, indian council of agricultural research; DAHD, department of animal husbandry and dairying.

The Figure 1 below illustrates the geographical distribution and list of BSL-3 laboratories across the country.

The biosafety and biosecurity measures laid down in guidelines the International Health Regulations by World Health Organization (WHO) (World Health Organization, 2005), Biosafety in Microbiological and Biomedical Laboratories (BMBL) by Centre for Disease Control and Prevention (CDC) (CDC, 2020) and Global Health Security Agenda (Global Health Security Agenda, 2020) provide strong frameworks for laboratories that specially deal with infectious disease hazards. The Indian guideline for the establishment and certfication of the BSL-3 containment facility was upgraded in 2024 projects the comprehensive guidance for the design, operation anf functionalization of BSL-3 laboratories (IBKP, 2024). Apart from this, a national Regulations and Guidelines for Reocminant DNA Research and Biocontainment was developed in 2017 by Department of Biotechnology (DBT), Government of India which would guide these BSL-3 laboratories for handling as well as for doing research on highly infectious pathogens primarily (DBTINDIA, 2017). Despite the elaborate biosafety regulatory framework in place in India, there should be an integrated and internalized system of assessment that helps the laboratories to assess their preparedness to operate, skill levels of the personnel, handling of samples, and reporting in accordance with the existing DBT and ICMR regulatory frameworks. The presented framework is a future-looking tool that seeks to enhance the performance of biosafety and biosecurity and enhance the process of coordination in the event of responding to the outbreak. We present the tool here, which is aimed at evaluating the preparedness of the BSL-3 laboratories to safe and rapid testing during the outbreak to make sure that the biosafety and biosecurity standards can be guaranteed and provide the efficient sample processing and reporting. The biosafety and biosecurity standards outlined in tool are based on certification guidelines of DBT and could be an additional internal assessment mechanism. They are supposed to support continuous preparedness and performance monitoring towards scope of prevailing certification procedures.

The questionnaire/checklist for the laboratory assessment for selected BSL-3 laboratories was developed after meticulous brainstorming sessions and co-ordination with a multidisciplinary team comprising of biosafety experts and specialists having in-depth knowledge of BSL-3/4 requirements and best practices, scientists with practical experience in laboratory operations and safety along with experts familiar with emergency protocols and response strategies. The final questionnaire/checklist was developed through iterative drafts and reviews, and by systematically addressing various aspects of laboratory operations, safety protocols, and equipment management so that it covers all relevant aspects of BSL-3 laboratory operations. The developed assessment tool was validated in four BSL-3 laboratories across different geographical locations in India.

India has an established regulatory framework of biosafety and biosecurity in the form of the Containment Guidelines (2017), The Certification Guidelines of BSL-3 Facilities (2024) and the Handbook of Institutional Biosafety Committees (2020) issued by the DBT. The present framework aims at enhancing these systems, by offering a systematic internal evaluation mechanism that facilitates timely testing and reporting of such occurrences in the event of public health emergency. This framework offers a systematic method of standardisation of biosafety and biosecurity assessment of the national network of BSL-3 laboratories in India.

## 2 Methodology

**FIGURE 2:**
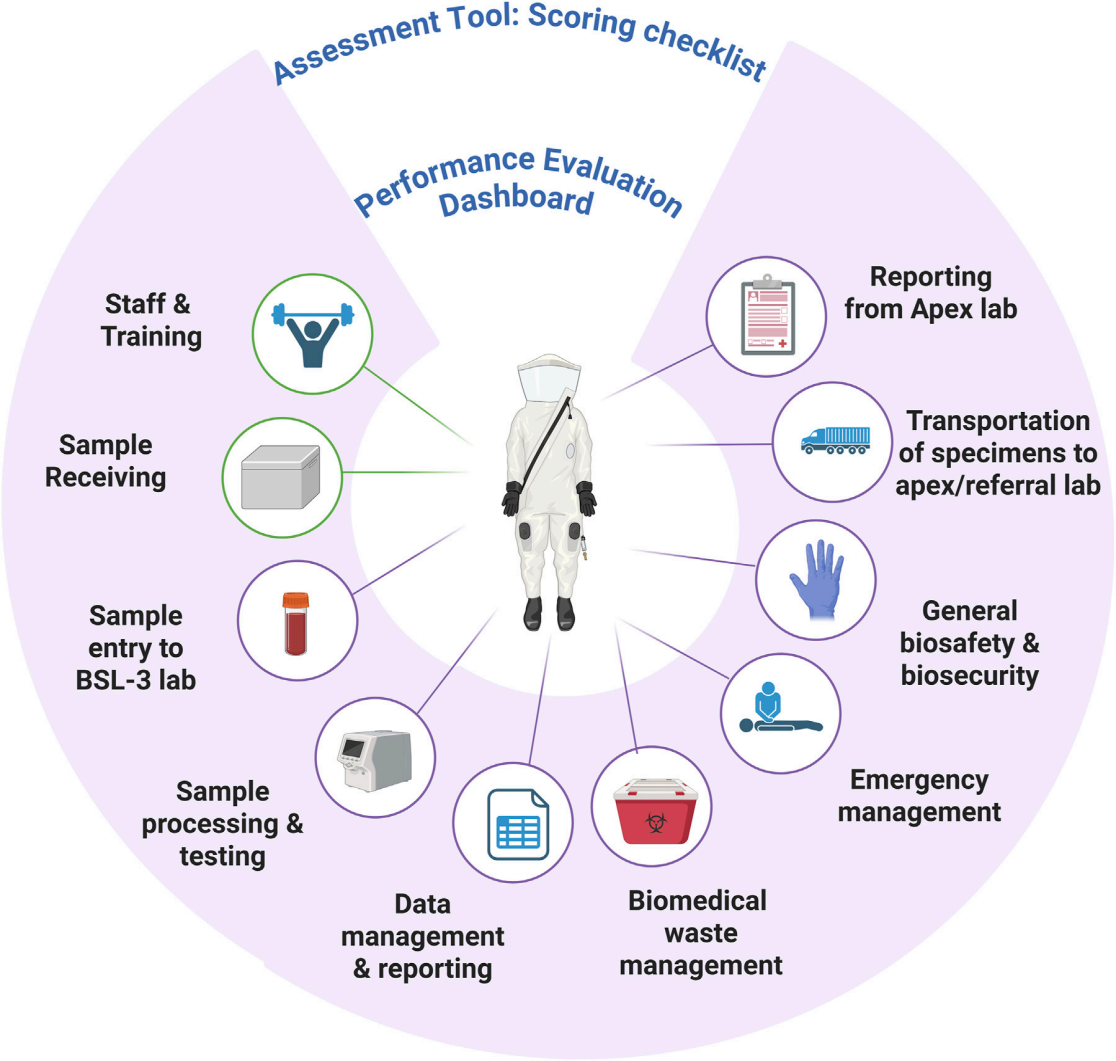
Framework for evaluating biosafety and biosecurity in BSL-3 laboratories.

All the BSL-3 facilities in India must be certified by the department of biotechnology (DBT) or by their own respective line ministry according to the Rules, 1989 under the environment (protection) act, 1986. The tool proposed is intended to complement this process by enabling the laboratories to self-assess themselves after a certain time to maintain compliance between certification cycles. The assessment tool leverages a structured scoring system organized into ten primary modules: staff and training, sample handling and transportation, sample receiving, sample transport inside the BSL-3 laboratory, sample processing and testing procedures, data management, biomedical waste management, emergency preparedness response, general biosafety and biosceurity, transportation of specimens and reporting of results (Figure 2). These components include detailed questions and scoring guides of the activities in each of the specified modules to assess compliance in various areas of laboratory operation. The tool can generate the score and show in a single picture the biosafety as well as biosecurity practices and compliance of the laboratories and highlight the gaps. The assessment tool has been created according to the Department of Biotechnology (DBT) National Guidelines for the Establishment and Certification of Biosafety Level-3 Containment Facilities (2024), making sure that all the criteria, scoring weightage, and procedural benchmarks were aligned with the national standards and consistent with other international standards and frameworks.

## 3 Details and scoring criterias of different modules for under framework of assessing BSL-3 laboratories

### 3.1 Staff and training

Biosafety measures specific to high containment laboratory include rules of standard hygiene and biosafety precautions, good laboratory practices, safety training on exposure to biohazardous substances, and disaster management. Using such criteria, this module assesses personnel/training insufficiencies likely to affect biosafety and biosecurity within the laboratory. The details are included in Table 1. This module evaluates the adequacy and preparedness of laboratory and laboratory personnel based on:

- Personnel records: Focuses on strength, qualification, skills including competencies and health records of both the employees and contractors.
- Training and certification: Training record is expressed in terms of scores derived from records in BSL-3 protocols such as Personal Protective Equipment (PPE) usage, spill and incident management along with emergency management.
- Infrastructure and capacity building measures: Evaluates the laboratory’s capability in ensuring adequate human resource training to operate the laboratory safely and without interruption. There would be constant need for refresher training to maintain the sustainability of the infrastructure.

**TABLE 1:** Scoring criteria for staff and training.

Sr. No	Steps	Sub-steps	Observations		Scoring
1	Details of the staff specifically trained for working in BSL-3 facility and support BSL-2	1.1 Suffiecient permanent staff [Number with designation]	Yes No	☐ ☐	
		1.2 ^a^Biosafety Officer [Permanent]	Yes No	☐ ☐	
		1.3 Sufficeient project staff [Number with designation	Yes No	☐ ☐	
		1.4 Sufficient engineering support [Number with designation]	Yes No	☐ ☐	
		1.5 Sufficient Multi-tasking staff [MTS] [Number]	Yes No	☐ ☐	
		1.6 Training record for working in BSL-3 laboratory	Yes No	☐ ☐	

^a^ Absence indicates the major non-conformities.

### 3.2 Sample receiving procedures

This module evaluates characteristics of sample receipt, transfer and documentation protocols, which is extremely pertinent to issues of biosafety. Adherence to the specific procedures can enable organization to manage its samples correctly and handle them appropriately to avoid any contamination or exposure risks within the facility or outside (Table 2).

- Sample receiving protocols: It assesses the performance of the lab, especially on the process of checking the labels, the sample chain of custody, and decontamination.
- Internal transport protocols: Examines staff compliance with proper transport procedures that are practiced inside the facility including the biosafety and biosecurity checks.
- Documentation: Checks that sample handling Standard Operating Procedures (SOPs) and the relevant training records exist.

**TABLE 2:** Scoring criteria for sample receiving procedures.

Sr. No	Steps	Sub-steps	Observations		Scoring
2	Sample Receiving	2.1 Prior intimation- electronic communication related to sample transport and testing [check email/text message]	Yes No	☐ ☐	
		2.2 Staff could verify chain of transport, documentation is complete and accurate	Yes No	☐ ☐	
		2.3 Staff could ensure proper labelling on the package [As per the Cat A and B shipment] (World Health Organization, 2021)	Yes No	☐ ☐	
		2.4 ^a^Staff could identify for leaks or breaches in the outer package and note the same in the sample receipt register	Yes No	☐ ☐	
		2.5 Staff could ensure the hassle free and coordinated receipt of the sample box	Yes No	☐ ☐	
		2.6 ^b^Availability of spill kit at sample receipt area	Yes No	☐ ☐	
		2.7 Staff followed the outer box disinfection procedure at defined area	Yes No	☐ ☐	
		2.8 Staff aware of the mechanism to address any leakage or breach in the condition of the boxes received	Yes No	☐ ☐	
		2.9 Staff has documented the receipt of samples (including condition, time, date, name of person and signature)	Yes No	☐ ☐	
		2.10 Availability of hand-held scanner (Security scanning device)	Yes No	☐ ☐	
		2.11 Scanner was used by staff to scan the specimen box	Yes No	☐ ☐	
		2.12 Documentations			
		a. Standard operating procedure (SOP) of sample receipt	Yes No	☐ ☐	
		b. SOP of sample transportation and triple packaging	Yes No	☐ ☐	
		c. SOP of spill management	Yes No	☐ ☐	
		d. Acceptance rejection criteria	Yes No	☐ ☐	
		Training records-			
		• SOP related (a,b,c,d)	Yes No	☐ ☐	
		• Spill management	Yes No	☐ ☐	
		• Personal protective equipment (PPE) donning and doffing while receiving the samples	Yes No	☐ ☐	
		• Biomedical waste (BMW) management	Yes No	☐ ☐	
		• Sample packaging and transportation	Yes No	☐ ☐	
		Records			
		• Sample receipt register	Yes No	☐ ☐	
		• Incident Report	Yes No	☐ ☐	

^a^ Absence indicates the major non-conformities.

^b^ Absence indicates the minor non-conformities.

### 3.3 Sample transport inside the BSL-3 facility

Sample processing is another key risk-priming zone in BSL-3 facilities. The exposure and release of pathogens is minimized by laboratories if there is adequate compliance with biosafety and biosecurity measures. This is a greatly weighted module as sample processing entails working closely with infectious specimens, something that requires strong biosafety measures (Table 3).

- Entry/Exit procedures: It tests compliance with entry/exit procedures with appropriate PPE, an emphasis on donning and doffing the personal protective gear and cautious working inside the containment facility.
- Working in Containment: Assesses the knowledge of staff on the working in BSL-3 facilities and compliance to good laboratory practices.

**TABLE 3:** Scoring criteria for sample transport inside the BSL-3 facility.

Sr. No	Steps	Sub-steps	Observations		Scoring
3	Sample transport to the BSL-3 facility	3.1 ^a^Availability of access control for the entry in BSL-3 facility	Yes No	☐ ☐	
		3.2 ^b^Staff has used the SS trolley to carry the specimen box to the BSL-3 facility	Yes No	☐ ☐	
		3.3 Staff used dynamic passbox/autoclave/airlock for the entry of the specimen box (depending on size of the specimen box)	Yes No	☐ ☐	
		3.4 Documentations			
		a. SOP of sample transport inside the facility	Yes No	☐ ☐	
		b. SOP for operation of Passbox	Yes No	☐ ☐	
		c. SOP for operation of Autoclave (clean cycle) if used for sample entry	Yes No	☐ ☐	
		d. Training records			
		• SOP related (a,b,c)	Yes No	☐ ☐	
		e. Records			
		• Authorized access control records	Yes No	☐ ☐	

^a^ Absence indicates the major non-conformities.

^b^ Absence indicates the minor non-conformities.

### 3.4 Sample aliquoting and testing

This module is crucial for assessing procedures where working with potentially infected samples is involved. It evaluates the skill and good laboratory practices of the staff, facility preparedness, and compliance with biosafety measures in the BSL-3 setup when doing sample aliquoting and testing (Table 4).

- Entry protocols and PPE: Observations relate to entry protocols of the staff, confirming compliance with SOPs for donning and doffing of the right PPE like coveralls and powered air purifying respirators (PAPRs). It is important to use PPEs appropriately to minimize contact with high risk pathogens.
- Facility equipment: In this regard, it confirms the existence and condition of equipment central to sample handling, including but not limited to biological safety cabinets and air handling units. To prevent cross contamination, the module guarantees that there are individual zones and air handling systems for viral/bacterial, human and animal samples.
- Aliquoting and sample inactivation: Observations of staff sample aliquoting practices include the following: SOP for sample handling; inactivation procedures such as heat or lysis buffer; and good laboratory practices. These processes are important in inactivating the specific pathogen before the test can be done.
- Testing procedures: Employees are assessed in elements such as ability to perform nucleic acid amplification test (NAAT), serology, sequencing, pathogen isolation etc. Adherents make observations on procedural compliance and data entry since documentation is a critical hallmark of traceability.
- Storage and sample transfer: Maintenance of sample storage and transfer after testing and to other areas is evaluated to determine if the samples are stored in the correct manner, for example, by use of dunk tanks for transfers and correct storage of aliquots in authorized freezers.
- Documentation: It focuses on checking training records, SOPs, and incident records for adequacy to cover all aspects of sample handling, testing and using the equipment.
- Power backup for critical equipment: To make sure that testing equipments are on a standby power system so as not to lose data in the middle of a test or even to spoil samples that need to be tested.

**TABLE 4:** Scoring criteria for sample aliquoting and testing.

Sr. No	Steps	Sub-steps	Observations		Scoring
4	Sample aliquoting and testing	4.1 Staff entered the BSL-3 facility as per the SOP (observe)	Yes No	☐ ☐	
		4.2 Staff donning the PPE (Coverall and PAPR) as per the SOP (observe)	Yes No	☐ ☐	
		4.3 ^a^Availability of separate room for handling virus and bacterial suspected pathogens	Yes No	☐ ☐	
		4.4 ^a^Availability of separate room for handling animal and human specimens	Yes No	☐ ☐	
		4.5 ^a^Availability of separate Air Handling Units for different rooms	Yes No	☐ ☐	
		4.6 Availability of biological safety cabinet (BSC) [Mention Type]	Yes No	☐ ☐	
		4.7 Staff uses the BSC to open the secondary container	Yes No	☐ ☐	
		4.8 Checked the Case Record Form (CRF)- Completeness and accuracy	Yes No	☐ ☐	
		4.9 Knows the mechanism for handling any discrepancies in CRF	Yes No	☐ ☐	
		4.10 Trained to read and understand the CRF of human origin	Yes No	☐ ☐	
		4.11 Trained to read and understand the CRF of animal origin	Yes No	☐ ☐	
		4.12 Staff is well aware of the procedure for sample acceptance and rejection- [Leakage/Labelling/cold chain]	Yes No	☐ ☐	
		4.13 Could perform the aliquoting based on the type of the specimens (consider good laboratory practices and biosafety)	Yes No	☐ ☐	
		4.14 Could perform the procedure for inactivation of the specimens [heat/dry bath or lysis buffer]	Yes No	☐ ☐	
		4.15 Staff has transported the samples for testing through Dunk Tank	Yes No	☐ ☐	
		4.16 Stored the remaining aliquot in freezer of BSL-3 facility as per the SOP	Yes No	☐ ☐	
		4.17 ^b^Staff doffed the PPE (Coverall and PAPR) as per the SOP (observe)	Yes No	☐ ☐	
		4.18 ^b^Tests performed as per the established protocol [Check the performance of the staff (GLP and Biosafety)]			
		a) NAAT	Yes No	☐ ☐	
		b) Serology	Yes No	☐ ☐	
		c) Agglutination assay	Yes No	☐ ☐	
		d) Neutralization assay	Yes No	☐ ☐	
		e) Isolation using *in-vitro* method	Yes No	☐ ☐	
		f) Isolation using *in-vivo* method	Yes No	☐ ☐	
		g) Sequencing	Yes No	☐ ☐	
		h) Others (specify)	Yes No	☐ ☐	
		4.19 Staff has recorded the raw data	Yes No	☐ ☐	
		4.20 ^a^The results are verified by the supervisor	Yes No	☐ ☐	
		4.21 ^b^Power back-up for the critical equipment	Yes No	☐ ☐	
		4.22 Documentations			
		a. SOP of entry-exit of personnel	Yes No	☐ ☐	
		b. SOP of donning and doffing of PPE [Coverall and Powered air purifying respirator (PAPR)]	Yes No	☐ ☐	
		c. SOP of working in BSC	Yes No	☐ ☐	
		d. SOP for spill management in BSC and outside BSC	Yes No	☐ ☐	
		e. SOP of sample aliquoting and storage	Yes No	☐ ☐	
		f. SOP of sample inactivation before testing in BSL-2 facility	Yes No	☐ ☐	
		g. SOP of specimen exit from BSL-3 laboratory	Yes No	☐ ☐	
		h. SOP of reagent aliquoting, storage and inventory management	Yes No	☐ ☐	
		i. Test related SOP available			
		• NAAT	Yes No	☐ ☐	
		• Serology	Yes No	☐ ☐	
		• Agglutination assay	Yes No	☐ ☐	
		• Neutralization assay	Yes No	☐ ☐	
		• Isolation using *in-vitro* method	Yes No	☐ ☐	
		• Isolation using *in-vivo* method	Yes No	☐ ☐	
		• Sequencing	Yes No	☐ ☐	
		• Others (specify)	Yes No	☐ ☐	
		• SOP of Incident reporting and mechanism	Yes No	☐ ☐	
		j. Training records			
		• SOP related (a-i)	Yes No	☐ ☐	
		• Spill management inside BSC	Yes No	☐ ☐	
		• BMW management	Yes No	☐ ☐	
		• Test related training of the staff	Yes No	☐ ☐	
		k. Records			
		• Sample details entry and recording	Yes No	☐ ☐	
		• Replacement schedule of quaternary ammonium compound (QAC) in dunk tank	Yes No	☐ ☐	
		• Competence/proficiency records of staff related to testing	Yes No	☐ ☐	
		• List of roles and responsibilities of the staff	Yes No	☐ ☐	
		• Incident reports	Yes No	☐ ☐	
		• Inventory records of samples	Yes No	☐ ☐	
		• Inventory records of reagents	Yes No	☐ ☐	
		• Calibration records of equipment			
		○ BSC	Yes No	☐ ☐	
		○ Real time PCR	Yes No	☐ ☐	
		○ PCR	Yes No	☐ ☐	
		○ Nucleic Acid Extractor	Yes No	☐ ☐	
		○ ELISA washer	Yes No	☐ ☐	
		○ ELISA Reader	Yes No	☐ ☐	
		○ Dry Bath	Yes No	☐ ☐	
		○ Centrifuge	Yes No	☐ ☐	
		○ Freezers (−80 C)	Yes No	☐ ☐	
		○ Freezers (−20 C)	Yes No	☐ ☐	
		○ Refrigerator (+4 C)	Yes No	☐ ☐	
		○ BOD Incubator	Yes No	☐ ☐	
		○ CO_2_ Incubator	Yes No	☐ ☐	
		○ Microscope	Yes No	☐ ☐	
		○ Sequencer	Yes No	☐ ☐	
		○ Micropipettes	Yes No	☐ ☐	
		○ UPS	Yes No	☐ ☐	
		• Annual Maintenance Contract record of the major installations, i.e., Autoclave, BLED tank, air handling units, air locks, pass box	Yes No	☐ ☐	
		• Annual shut down and maintenance report	Yes No	☐ ☐	
		• Test related records	Yes No	☐ ☐	

^a^ Absence indicates the major non-conformities.

^b^ Absence indicates the minor non-conformities.

### 3.5 Reporting and data management

The biosafety and biosecurity procedure entails measures in protection and management and reporting of data. This module assures that data is handled with propriety, as well as standardize documentation practices are followed throughout the laboratory (Table 5).

- Secure data storage: Score the facilities based on the availability of password protected computer and laboratory data management through malware protected system.
- Data confidentiality: Reviews lab on status of compliance to polices on secrecy of information and authorized personnel to access them.
- Data entry and reporting: Ensure the integrity of the lab in displaying the method of recording and reporting of results.

**TABLE 5:** Scoring criteria for reporting and data management.

Sr. No	Steps	Sub-steps	Observations		Scoring
5	Reporting and Data Management	5.1 Staff recorded that data and prepared the report in the format	Yes No	☐ ☐	
		5.2 ^a^Availability of password protected computer	Yes No	☐ ☐	
		5.3 ^b^Availability of secured connections/Malware protection	Yes No	☐ ☐	
		5.4 Documentation			
		a) SOP of data management	Yes No	☐ ☐	
		b) SOP of reporting test results	Yes No	☐ ☐	
		c) Institutional policy for data management and cyber security	Yes No	☐ ☐	
		d) Training records			
		• SOP related	Yes No	☐ ☐	
		• Recording and reporting of test results	Yes No	☐ ☐	
		• Cyber security training			
		e) Records-			
		• Reports [Positive/negative]	Yes No	☐ ☐	
		• CRF files	Yes No	☐ ☐	
		• Declaration of Confidentiality	Yes No	☐ ☐	
		• List of authorized personnel to access the data	Yes No	☐ ☐	
		• Data sheets	Yes No	☐ ☐	

^a^ Absence indicates the major non-conformities.

^b^ Absence indicates the minor non-conformities.

### 3.6 Biomedical waste management

It is particularly important to manage biomedical waste appropriately in order to reduce the risk of infection to the waste handlers. Relative to other forms of biomedical waste, this module seeks to engage responsible management of risks associated with biohazardous waste through compliance with laws (Table 6).

- Biomedical waste disposal systems: Assesses procedures and effective functioning of autoclave and biological liquid effluent decontamination (BLED) tank.
- Training: Checks whether biomedical waste management staff knows their standard operating procedures (SOPs) and whether they have received proper training.
- Documentation: Examines logbooks of autoclaves as well as BLED tanks.

**TABLE 6:** Scoring criteria for biomedical waste management.

Sr. No	Steps	Sub-steps	Observations		Scoring
6	Biomedical Waste Management	6.1 ^a^Availability of functional autoclave	Yes No	☐ ☐	
		6.2 ^b^Check for the performance of autoclave operator	Yes No	☐ ☐	
		6.3 ^a^Availability of functional biological liquid effluent decontamination (BLED) tank	Yes No	☐ ☐	
		6.4 ^b^Check for the performance of BLED tank operator	Yes No	☐ ☐	
		6.5 ^b^Date and time of last fumigation of BSL-3 facility	Yes No	☐ ☐	
		6.6 Documentations			
		a) SOP for operation of autoclave	Yes No	☐ ☐	
		b) SOP for operation of BLED	Yes No	☐ ☐	
		c) SOP of Fumigation and decontamination	Yes No	☐ ☐	
		Training records-			
		a) SOP related (a,b,c)	Yes No	☐ ☐	
		b) Record of staff for the operation of autoclave	Yes No	☐ ☐	
		c) Record of staff for the operation of BLED tank	Yes No	☐ ☐	
		Records-			
		• Validation records of Autoclave	Yes No	☐ ☐	
		• Validation record of BLED	Yes No	☐ ☐	
		• Fumigation records	Yes No	☐ ☐	
		• List of authorized agencies for BMW	Yes No	☐ ☐	
		• Records of different categories of waste generated per month	Yes No	☐ ☐	
		• AMC/CMC records of Autoclave and BLED	Yes No	☐ ☐	

^a^ Absence indicates the major non-conformities.

^b^ Absence indicates the minor non-conformities.

### 3.7 Emergency management

Some aspects of emergency preparedness entail the provision of items such as firefighting equipments, first aid kits and automated external defibrillators (AEDs) and ensuring that all staff undergoes rigorous training and sensitization in case of an emergency. Hazard management in laboratories includes an effective emergency preparation module that enhances lab resilience in case of adversity (Table 7).

- Emergency equipment: Checks that all necessary emergency tools are on hand and easily visible in the laboratory.
- Evacuation plans and protocols: Checks how staff is acquainted with the existence and location of the available main exits and how they react in case of an emergency.
- Incident documentation: Confirms the existence of incident reporting SOPs and the maintenance logs for emergency equipment and whether they are updated periodically.

**TABLE 7:** Scoring criteria for emergency management.

Sr. No	Steps	Sub-steps	Observations		Scoring
7	Emergency Management	7.1 ^a^Availability of smoke detectors	Yes No	☐ ☐	
		7.2 ^a^Availability of Fire Extinguishers inside BSL-3 facility	Yes No	☐ ☐	
		7.3 Availability of Fire Extinguishers in BSL-2 facility	Yes No	☐ ☐	
		7.4 ^b^Staff could easily access and operate the Fire extinguisher	Yes No	☐ ☐	
		7.5 Tagging of Fire Extinguisher with re-fill dates	Yes No	☐ ☐	
		7.6 Availability of Fire Blankets	Yes No	☐ ☐	
		7.7 ^b^Staff has awareness of Emergency Exit pathways and signage	Yes No	☐ ☐	
		7.8 Staff is aware of the places where the details of contact numbers for emergency management are displayed	Yes No	☐ ☐	
		7.9 Availability of automated external defibrillator (AED)	Yes No	☐ ☐	
		7.10 ^b^Staff has been trained for cardiopulmonary resuscitation (CPR) and AED operation	Yes No	☐ ☐	
		7.11 Availability of First Aid Kit	Yes No	☐ ☐	
		7.12 Staff trained to provide general first aid	Yes No	☐ ☐	
		7.13 Documentations			
		a) SOP of Emergency Responses			
		• Fire	Yes No	☐ ☐	
		• Spill	Yes No	☐ ☐	
		• Splashes in eye	Yes No	☐ ☐	
		• Heart attacks/seizures/Stroke	Yes No	☐ ☐	
		• Fall/Fractures	Yes No	☐ ☐	
		• Burn injuries- Chemical Spill	Yes No	☐ ☐	
		• Needle stick injuries	Yes No	☐ ☐	
		• Inhalation of toxic fumes/infectious aerosols, etc.	Yes No	☐ ☐	
		• Injuries while handling animals	Yes No	☐ ☐	
		• Flood	Yes No	☐ ☐	
		• Tornado	Yes No	☐ ☐	
		• Lightning	Yes No	☐ ☐	
		• Earthquakes	Yes No	☐ ☐	
		• Power outage	Yes No	☐ ☐	
		• Hostages/Active shooter, Bomb threat	Yes No	☐ ☐	
		b) Training records			
		• SOP of Emergency Responses	Yes No	☐ ☐	
		• Fire management- Operation of Fire extinguisher and water Hoses	Yes No	☐ ☐	
		• Use of AED and CPR	Yes No	☐ ☐	
		c) Records			
		• Incident/Accident/Event reporting	Yes No	☐ ☐	
		• Refill of Fire-extinguishers	Yes No	☐ ☐	
		• AMC of AED	Yes No	☐ ☐	
		• List of hospitals authorized for clinical management	Yes No	☐ ☐	
		• Details of nearest Fire Brigade	Yes No	☐ ☐	
		• Details of nearest police station	Yes No	☐ ☐	

^a^ Absence indicates the major non-conformities.

^b^ Absence indicates the minor non-conformities.

### 3.8 General biosafety and biosecurity

General biosafety practices draw and establish compliance to general laboratory safety standards, validation and health precaution. This module evaluates general safety and biosecurity measures related to laboratory personnel’s protection in their line of duty (Table 8).

- Facility certification and signage: Confirmation about the validation of BSL-3 facilities and the ease by which biohazard signs can be seen.
- Biosecurity risk assessment: Review of the laboratory biosecurity risk assessment based on personal, physical, inventory security, information and cyberscurity.
- Vaccination and health monitoring: Scoring based on records of staff vaccination and preventive health check-up.
- Pest control and general hygiene: Check pest control and hygiene measures, improves the environment management.
- Risk assessment documents: Highlighting the risk involved and mitigation measures

**TABLE 8:** Scoring criteria for general biosafety and biosecurity.

Sr. No	Steps	Sub-steps	Observations		Scoring
8	General Biosafety and Biosecurity of the BSL-3 laboratory and support BSL-2 laboratory	8.1 ^a^Certification of the BSL-3 facility	Yes No	☐ ☐	
		8.2 ^b^Date of last certification and validity [Mention]- Is it displayed?	Yes No	☐ ☐	
		8.3 Adequate display of the Biohazard signages	Yes No	☐ ☐	
		8.4 Posted information must include: the laboratory’s biosafety level, supervisor’s name (or other responsible personnel), telephone number	Yes No	☐ ☐	
		8.5 Staff is aware of an effective integrated pest (insect and rodent) management program is required	Yes No	☐ ☐	
		8.6 ^b^Dates available of last pest control done in previous year or months	Yes No	☐ ☐	
		8.7 Pre-work initiation sera samples of the staff available as bio-bank	Yes No	☐ ☐	
		8.8 Biosecurity risk assessment			
		a) Personal security			
		• Does the staff have knowledge about the biosecurity aspects and risks of the laboratory work?	Yes No	☐ ☐	
		• Does laboratory In-Charge conduct frequent assessment of human factors that may affect biosecurity (for example, work stress, dismissals, disagreements in a team, jealousy, financial debt, drug abuse)?	Yes No	☐ ☐	
		• Have personnel with access to high-consequence material undergone a background investigation and been considered suitable before being allowed access to the material?	Yes No	☐ ☐	
		• Are personnel with access subject to periodic or ongoing scrutiny?	Yes No	☐ ☐	
		• Is there a process for self- and peer-reporting of incidents or behaviours of concern?	Yes No	☐ ☐	
		b) ^b^Physical security			
		• Is attendance of personnel in biosecurity-relevant areas of the facility regulated, monitored and/or recorded?	Yes No	☐ ☐	
		• Is the facility monitored using a video surveillance system or similar?	Yes No	☐ ☐	
		• Are there any intruder alarm systems in place?	Yes No	☐ ☐	
		c) ^a^Inventory security			
		• Is an inventory security system in place for high-consequence materials?”	Yes No	☐ ☐	
		• Are regular audits performed on the inventory system?	Yes No	☐ ☐	
		d) ^a^Information security and cybersecurity			
		• Is access to laboratory information systems restricted to authorized personnel only?	Yes No	☐ ☐	
		• Are USB ports, external drives, or wireless connectivity restricted or monitored in the BSL-3 lab systems?	Yes No	☐ ☐	
		• Are regular backups of critical data performed and stored securely in an offsite or encrypted location?	Yes No	☐ ☐	
		• Is there a documented cybersecurity incident response plan specific to BSL-3 data and systems?	Yes No	☐ ☐	
		• Do all BSL-3 personnel receive periodic training on information security and cybersecurity?	Yes No	☐ ☐	
		8.9 Documentations			
		a) Records			
		• Vaccination records of staff	Yes No	☐ ☐	
		• Records of antibody titres where possible	Yes No	☐ ☐	
		• Annual Preventive Medical Examination including psychological assessment	Yes No	☐ ☐	
		• General biosafety and good laboratory/microbiological practices training records	Yes No	☐ ☐	
		• Training records for small animal handling	Yes No	☐ ☐	
		b) Risk Assessment documents			
		• Major installations	Yes No	☐ ☐	
		• Laboratory work flow	Yes No	☐ ☐	
		• Biosecurity (Personal, physical, inventory, information and cybersecurity)	Yes No	☐ ☐	
		• Emergency management	Yes No	☐ ☐	
		• Laboratory equipment	Yes No	☐ ☐	

^a^ Absence indicates the major non-conformities.

^b^ Absence indicates the minor non-conformities.

### 3.9 Transportation of specimens from BSL-3 laboratory to apex/referral laboratory

Transporting specimens from a BSL-3 laboratory to an apex/referral laboratory requires strict adherence to safety protocols to prevent the release of potentially dangerous pathogens. The procedures need to follow is as per the WHO transportation guidelines (World Health Organization, 2021) for shipment of clinical specimens. The documentation process, triple packaging methods, chain of custody plays a integral role for this process (Table 9).

**TABLE 9:** Scoring criteria for transportation of specimens from BSL-3 laboratory to apex/referral laboratory.

Sr. No	Steps	Sub-steps	Observations		Scoring
9	Transportation of specimens from BSL-3 laboratory to apex/referral laboratory	9.1 Inclusion plan during aliquoting itself- one aliquot of each specimens for immediate transportation	Yes No	☐ ☐	
		9.2 ^a^Proper labels for all the aliquots- Pre-labelled tubes ready inside BSL-3 lab	Yes No	☐ ☐	
		9.3 ^b^Contacted the courier agency immediately and arranged the shipment on same day	Yes No	☐ ☐	
		9.4 ^b^Contacted the referral/apex lab and provided the information for the transportation	Yes No	☐ ☐	
		9.5 After aliquoting inside BSL-3, the primary receptacles were surface disinfected inside the BSC	Yes No	☐ ☐	
		9.6 Gloves were changed before placing the primary receptacle into secondary receptacles	Yes No	☐ ☐	
		9.7 ^b^Appropriate absorbent materials were placed between primary and secondary receptacles	Yes No	☐ ☐	
		9.8 ^b^Secondary receptacles were properly surface disinfected before taking out of the BSC	Yes No	☐ ☐	
		9.9 The secondary receptacles were placed in biohazard bag and sealed	Yes No	☐ ☐	
		9.10 The sealed bag with secondary receptacles were immediately taken through Dunk Tank	Yes No	☐ ☐	
		9.11 One team outside was ready with the tertiary container with appropriate labels and forms	Yes No	☐ ☐	
		9.12 ^b^Emailed the Case Record Form scanned copies to the referral/apex laboratory	Yes No	☐ ☐	
		9.13 Emergency contact numbers and appropriate address mentioned in the outer box	Yes No	☐ ☐	
		9.14 Dry ice shipment could be arranged	Yes No	☐ ☐	
		9.15 Flight details discussed with courier agencies and informed to the apex/referral laboratory [referral lab/apex lab should receive the samples within 24 h]	Yes No	☐ ☐	
		9.16 Documentations			
		a) Records			
		• SOP for sample exit from BSL-3 laboratories for transportation	Yes No	☐ ☐	
		• Training records of staff on triple packaging and transportation	Yes No	☐ ☐	

^a^ Absence indicates the major non-conformities.

^b^ Absence indicates the minor non-conformities.

### 3.10 Reporting

The timely results from apex laboratory would play a crucial role in early detection of the outbreak and subsequent containment measures (Table 10). The validated report ensures reliability.

**TABLE 10:** Scoring criteria for Report from apex/referral laboratory.

Sr. No	Steps	Sub-steps	Observations		Scoring
10	Report from apex/referral laboratories	10.1 Responsible person defined by the laboratory for the follow up of the report from apex/referral laboratory	Yes No	☐ ☐	
		10.2 ^a^Apex/referral laboratory has shared the report within 24 h of receipt of samples	Yes No	☐ ☐	
		10.3 Documentations			
		a) Records			
		• Test report from the apex/referral laboratory	Yes No	☐ ☐	

^a^ Absence indicates the major non-conformities.

## 4 Final score interpretation and compliance categories

To enable standardized performance benchmarking, each item in the checklist is scored as “Yes” = 1 and “No” = 0, and module-wise scores are summed. Each of the 10 modules contributes a cumulative score based on the number of applicable indicators. The final laboratory score is the sum of “Yes” responses across all modules. For indicators deemed non-applicable (NA) based on the nature of the facility (e.g., animal-specific protocols not relevant for human labs), the scorers mark them as “NA” and these items are excluded from the total score computation. Based on the percentage of total applicable points achieved, laboratories can be categorized into one of the following compliance levels:

- Fully Compliant (≥90%): Strong performance across all domains; minimal or no corrective action needed.
- Partially Compliant (61%–89%): Some deficiencies identified; targeted improvements required in specific modules.
- Non-Compliant (<60%): Significant gaps in biosafety and biosecurity; immediate corrective action and oversight needed.

This structured scoring approach ensures transparency, reproducibility, and clear prioritization for corrective action across the BSL-3 laboratory network.

Whenever any lapses or deviations are detected under self-assessment, the laboratories should promptly report the same to their respective line ministry and the Review Committee on Genetic Manipulation (RCGM), DBT, to take the necessary corrective action.

## 5 Discussion

With continuously increasing numbers of high containment laboratories across various parts of the world, it is imperative to have a standard tool for assessing the core competencies, standard operating procedures, staff skillset, sample archival and disposal and report sharing processes. To the best of our knowledge, till date, no such tool is available in public domain. In view of this, we have developed a simple yet innovative tool for comprehensive assessment of various essential parameters of high containment laboratories. While developing this tool we have considered various reference points including the WHO Laboratory Biosafety Manual (World Health Organization, 2020), WHO Laboratory biosecurity guidance (World Health Organization, 2024) and BMBL (CDC, 2020). The scoring and assessment criteria under this framework are aligned with the DBT National Guidelines of BSL-3 containment facilities so that it will be credible and consistent across the laboratories of various fields like health, animal, and environmental sectors. This tool underwent piloting at four BSL-3 facilities across India, encompassing diverse geographical regions and operational mandates, including research, diagnostics, and public health initiatives pertinent to human health. The laboratories involved in testing and validating the tool were: the All India Institute of Medical Sciences (AIIMS), Bhopal, Madhya Pradesh; AIIMS Jodhpur, Rajasthan; the Regional Medical Research Centre, Dibrugarh, Assam; and the Jawaharlal Institute of Postgraduate Medical Education and Research, Puducherry. This varied selection of sites enhanced the assessment of the tool’s generalizability across different laboratory types and facilitated the objective identification of relevant non-compliances. The tool provides an opportunity to laboratory managers and auditors to readily assess the gaps and limitations in high containment laboratories and quickly understand the areas where improvements are needed, follow-up actions to be taken by lab managers and areas of focus for enhancing performance.

One important advantage of this tool is the possibility of each of the sections to be employed separately while if all of them need to be used, the full assessment is available. This flexibility is effective in particular in conducting targeted assessments when the laboratory is experiencing specific limitations. Further, application of this tool repeatedly to assess the performance of BSL-3 laboratories will help in continuous improvement of each work stream and quick redress of the gaps.

India has a very well-developed and well-regulated regulatory framework on biosafety, biosecurity, which is regulated under the Environment (Protection) Act, 1986, the Rules, 1989, and the Department of Biotechnology (DBT) Containment Guidelines (2017) and Certification Guidelines of BSL-3 Facilities (2024). With the expansion of BSL-3 laboratories mandate for testing samples from various domains including human, animal and environmental samples, it is critical to ensure the high level of functionality of these labs. Though this assessment tool has been designed considering its deployment in laboratories in India, it has the potential to be tweaked and deployed globally as well. The tool can be used for assessment by independent reviewers or the laboratory staff themselves to identify the gaps and bridge them appropriately. In response to the current lack of a national regulatory mechanism for containment laboratories in India, an advisory and review committee has been formed in November 2024. An advisory and review committee was formed and tasked with overseeing the 22 BSL-3 laboratories within the NOHM network, thereby strengthening biosafety and biosecurity measures nationwide. This tool will help in identifying the inadequacies in various adjacent verticals of BSL-3 laboratories in a quick and objective way and give insights on specific actions required. Repeated deployment of the tool in routine practice might result into enhanced biosafety practices thereby enhancing the capacity of countries to respond effectively to public health emergencies related to high-risk pathogens and biosecurity threats. The suggested framework is supposed to supplement these current systems as it is supposed to be used as a self-assessment tool and preparedness enhancement tool in national activities to ensure high levels of biosafety and biosecurity. The self-assessment and periodic review provisions included in this tool are introduced to supplement the process of DBT certification to avoid duplication and regulatory coherence. The outputs obtained in the framework can be incorporated into the database of national certification of DBT, thus, contributing to internal quality control and external regulatory control.

## Data Availability

The original contributions presented in the study are included in the article/supplementary material, further inquiries can be directed to the corresponding authors.

## Members of the National network of BSL-3 laboratories

Abdul Arif Khan (ICMR-National Institute of Translational Virology and AIDS Research, Pune, Maharashtra), Anamika Mishra (ICAR-National Institute of High Security Animal Diseases, Bhopal, Madhya Pradesh), Aniket Sanyal (ICAR-National Institute of High Security Animal Diseases, Bhopal, Madhya Pradesh), Arjun Guha (Institute For Stem Cell Science and Regenerative Medicine, Bengaluru, Karnataka), Ashwani Kumar (CSIR-Institute of Microbial Technology, Chandigarh), Ashwin. A. Raut (ICAR-National Institute of High Security Animal Diseases, Bhopal, Madhya Pradesh), B. Anukumar (ICMR-National Institute of Virology, field unit, Kerala), Biswa Borkakoty (ICMR-Regional Medical Research Centre, Dibrugarh, Assam), Debasis Biswas (All India Institute of Medical Sciences, Bhopal, Madhya Pradesh), Dhiraj Kumar (International Centre For Genetic Engineering and Biotechnology, New Delhi), Gaurav Kumar Sharma (Centre for Animal Disease Research and Diagnosis, Bareilly, Uttar Pradesh), Gulam H. Syed (Institute of Life Sciences, Bhubaneswar, Odisha), Harisankar Singha (ICAR-National Centre for Veterinary Type Cultures (NCVTC)/National Research Centre on Equines, Hisar, Haryana), Harshad V. Murugkar (ICAR-National Institute of High Security Animal Diseases, Bhopal, Madhya Pradesh), Jayati Mullick (ICMR-National Institute of Virology, Pune, Maharashtra), Radha Kanta Ratho (Post Graduate Institute of Medical Education & Research, Chandigarh), Rahul Dhodapkar (Jawaharlal Institute of Postgraduate Medical Education and Research, Puducherry), Rajeev Ranjan (ICAR-National Institute on Foot and Mouth Disease, Bhubaneswar, Odisha) Rajeev Singh (ICMR-Regional Medical Research Centre, Gorakhpur, Uttar Pradesh), Rajesh Chandramohanadas (BRIC-Rajiv Gandhi Centre for Biotechnology, Thiruvananthapuram, Kerala), Ramandeep Singh (Translational Health Science and Technology Institute, Faridabad, Haryana), Ravishekhar Gadepalli (All India Institute of Medical Sciences, Jodhpur, Rajasthan), Sandeep Kumar Singh (Chaudhary Charan Singh National Institute of Animal Health Baghpat, Uttar Pradesh), Santosh Chauhan (Centre for Cellular and Molecular Biology, Hyderabad, Telangana), Vijaya Lakshmi Nag (All India Institute of Medical Sciences, Jodhpur, Rajasthan), Vinay K. Nandicoori (Centre for Cellular and Molecular Biology, Hyderabad, Telangana).
